# Dry Needling of LI4 and TE5 Acupuncture Points on Wrist Flexor Spasticity in Stroke: A Case Report

**DOI:** 10.1155/crnm/2219978

**Published:** 2025-10-18

**Authors:** Najmeh Nazari, Noureddin Nakhostin Ansari, Pablo Herrero, Roshanak Honarpisheh, Zahra Mohammadi, Soofia Naghdi

**Affiliations:** ^1^Department of Physiotherapy, School of Rehabilitation, Tehran University of Medical Sciences, Tehran, Iran; ^2^Research Center for War-Affected People, Tehran University of Medical Sciences, Tehran, Iran; ^3^Faculty of Health Sciences, IIS Aragon, University of Zaragoza, Zaragoza, Spain; ^4^Neuromusculoskeletal Research Center, Iran University of Medical Sciences, Tehran, Iran

**Keywords:** acupuncture, case report, dry needling, motor function, spasticity, stroke

## Abstract

Spasticity is one of the most common serious complications after stroke. Dry needling (DN) has been used to improve spasticity and motor function in patients poststroke. This case report aimed to present the DN effects of LI4 and TE5 acupuncture points on wrist flexor spasticity in a patient with stroke. The patient was a 57-year-old man with a 5-year history of right hemiplegia poststroke. DN was applied on LI4 and TE5 for three sessions, every other day, and each point for 1 minute. The patient was assessed before (T0), after 3 sessions of DN (T1), and after first (T2) and sixth weeks (T3) after the last session. After DN, wrist flexor spasticity decreased from “2” to “0” according to the Modified Modified Ashworth Scale and improvement remained at T2 and T3. The wrist active range of motion (ROM) significantly improved from 0° at T0 to 10° at T1, 45° at T2, and 35° at T3 follow-up. The patient showed improvements in wrist passive ROM from 75° at T0 to 82° at T1, 90° at T2, and 80° at T3. The total motor score of Fugl-Meyer assessment demonstrated small improvements (38 points at T0 to 45 at T2, again 38 at T3). Three sessions of DN at LI4 and TE5 exhibited positive effects on spasticity and wrist ROM in a patient with stroke. Further investigation using the DN technique on acupoints in stroke patients with spasticity is warranted.

## 1. Introduction

Stroke is the first among the top conditions affecting the nervous system with the highest age-standardized disability-adjusted life-years (DALYs) in 2021 [1]. An updated estimate of the stroke burden shows a significant increase from 1990 to 2021, with stroke becoming the fourth leading cause of DALYs in 2021, at 160.5 million (147.8–171.6) [2]. Spasticity is a common and serious complication affecting 60% of patients after stroke [3]. Spasticity is typically defined as a velocity-dependent increase in muscle tone resulting from hyperexcitability of the stretch reflex [4]. However, a new definition of poststroke spasticity suggests a more comprehensive neurophysiological model, describing it as a velocity- and muscle length-dependent increase in resistance to muscle stretch caused by hyperexcitable descending excitatory brainstem pathways [5]. The primary lesion leading to spasticity is a neural mechanism, although there are also structural and mechanical changes in the spastic muscles [6, 7]. Substantial direct costs related to stroke survivors with spasticity are 4 times higher than patients without spasticity [8].

Muscle spasticity after stroke is accompanied by other motor impairments such as weakness, loss of dexterity, abnormal limb posture, and lack of coordination. Disabling spasticity appears to be more common in the elbow, wrist, and ankle following stroke, which leads to significant functional limitations [9]. There is a wide range of techniques for spasticity management which includes invasive and noninvasive approaches. Antispastic medications and physiotherapy are routinely used for spasticity.

Dry needling (DN) is a physiotherapy intervention that uses a thin monofilament needle to penetrate the skin and stimulates the underlying tissues. DN is an effective technique to improve spasticity and motor function in patients poststroke [10, 11].

Acupuncture is an intervention of Chinese medicine used to manage spasticity [12, 13]. There are structural changes in spastic muscle and neural hyperexcitability after stroke. Acupuncture may reduce spasticity and improve the structure of spastic muscles [14, 15]. We selected the Hegu (LI4) and Waiguan (TE5) acupoints due to their common use in reducing upper limb spasticity [13]. This case study describes the DN technique applied to both LI4 and TE5 in a patient with stroke and wrist flexor spasticity. The novelty of the study lies in the use of two traditional acupoints for the application of the deep DN technique. The rationale for selecting this combination of points was to enhance the therapeutic effects.

## 2. Case Presentation

The patient was a 57-year-old man who had suffered from right-sided hemiplegia following an ischemic stroke for five years. He was also right-hand dominant. The patient was alert and had an independent gait. His medical history included diabetes for 10 years and smoking for more than 30 years. He had undergone 30 sessions of routine physiotherapy after the stroke, which led to only limited improvement in right-hand function, one of his major concerns. The patient was not taking any medications that could influence spasticity, such as muscle relaxants or botulinum toxin. Furthermore, considering the potential risk of bruising or bleeding associated with DN, the patient was specifically interviewed to ensure he was not on anticoagulants. In other words, there were no contraindications to DN.

Demographic variables were first collected at baseline. The physical assessment indicated a Grade 2 of spasticity in the right wrist flexor muscles as measured with the Persian version of the Modified Modified Ashworth Scale (MMAS) [16]. The MMAS grades the spasticity level from “0” to “4” (Table 1). We chose to use the MMAS, as it is considered a more reliable tool than the original Ashworth scale and the modified Ashworth Scale [17]. Given the advancements—such as the removal of Grade 1+ from the MAS and the redefinition of Grade 2—the MMAS is recommended as a more appropriate measure in similar studies [17]. The assessor was a physiotherapist who was blinded to the case study and the type of intervention.

The Fugl-Meyer scale was used to assess the recovery of the shoulder, elbow, forearm, wrist, and hand movement. It consists of 33 upper extremity motor function items with total scores from 0 to 66. Each item is scored on an ordinal scale from 0 to 2 as follows: 0 = *cannot perform*, 1 = *performs partially*, and 2 = *performs fully*. The Fugl-Meyer demonstrates excellent reliability and validity when applied in clinical and research settings [18, 19]. The Fugl-Meyer motor domain scores assessed in this patient with stroke were upper extremity 23/36, wrist 0/10, hand 10/14, and coordination/speed component 5/6.

A standard goniometer was used (Baseline Plastic 360° ISOM, Goniometer 6″, 3B Scientific, Hungary) to measure the active and passive range of motion (ROM) of the wrist extension. The measurement was performed in sitting position with the elbow at 90° flexion and the forearm in the mid-position on a supporting surface. Clinical outcomes assessed before (T0) and immediately after 3 treatment sessions (T1), and at follow-up visits conducted in Week 1 after (T2) and Week 6 after the end of the third treatment session (T3).

**Figure 1:**
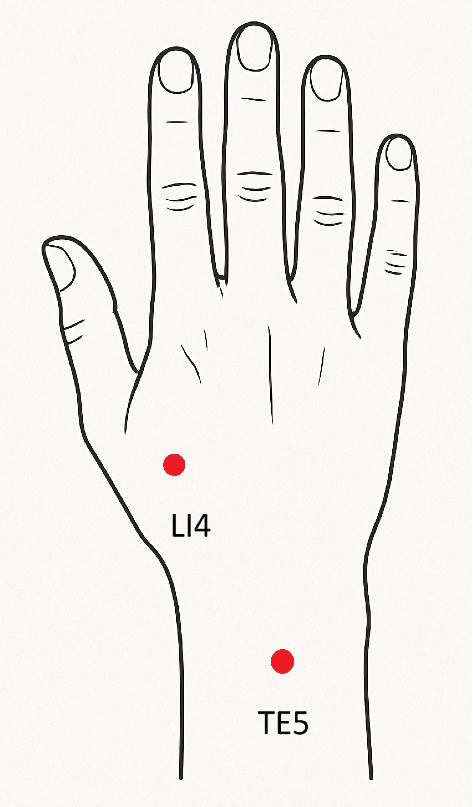
Acupoints used for dry needling. LI4 on the dorsal of the hand, at the angle between the first and second metacarpals. The TE5 is 2 cun above the transverse crease of the dorsum of the wrist, between the radius and ulnar.

The procedure was performed at the Neurophysiotherapy Clinic, School of Rehabilitation, Tehran University of Medical Sciences, Iran. Before the study procedure, the patient signed the informed consent. Three sessions of DN were performed every other day during 1 week. An experienced and certified physiotherapist applied the technique. Two acupuncture needles (size: 0.25 × 25 mm, DongBang AcuPrime Ltd, Korea) were used in LI4 and TE5 [20]. The procedure consisted of 1 minute of deep DN for each point, using a cone-shaped, fast-in and fast-out technique. With the forearm in pronation, LI4 was needled on the dorsal region of the hand, at the angle between the first and second metacarpals. In the same position, the *TE5* was needled 2 cun (unit of length equals to the width of the interphalangeal joint of the thumb) above the transverse crease of the dorsum of the wrist, between the radius and ulnar [20]. A recent review suggested the LI4 and TE5 are the most used points for managing the upper-limb and wrist spasticity [13].  Figure 1 depicts the points used for DN.

All outcomes improved after DN (Table 2). Significant improvements were noted in spasticity (grade “2” to “0”) at T1, as well as at T3. Before DN at T0, the wrist AROM was 0° and improved immediately after DN at T1 to 10° and 45°at T2. Furthermore, the improvements in the PROM were observed at both T1 and follow-up time points. FMA-wrist improved from 0 at T0 to 5 at T2. The patient reported pain and discomfort during DN, especially with the LI4 point.

## 3. Discussion

This case report describes the successful use of DN on spasticity when applied to the acupuncture points of LI4 and TE5 in the management of a patient with a chronic stroke. In our case study, DN was performed at acupuncture points using a manipulation technique with a fast-in, fast-out approach, rather than targeting trigger points or motor points. This approach was chosen to specifically assess the effects of DN when applied to the LI4 and TE5 acupuncture points. To the best of our knowledge, this is the first report on the use of DN at acupuncture points; therefore, no previous studies are available for comparison with our findings.

Previous investigations have demonstrated the positive effects of DN on spasticity in patients with stroke [10, 21]. The improvements in spasticity might be from the better performance of the gamma motor system post-DN [21]. A recent systematic review of 10 studies to evaluate the efficiency of acupuncture in poststroke spasticity concluded that acupuncture is a complementary option in improving spasticity and quality of life after stroke [22]. Clinical trials have shown that acupuncture can be a useful supplement to improve poststroke impairment and motor ability [23, 24]. A systematic review and meta-analysis of eight studies involving 6431 individuals concluded that acupuncture may be recommended as adjuvant therapy for poststroke spasticity [25]. Additionally, it is possible that the observed improvements in spasticity could be related to a decrease in the excitability of the spinal hyperreflexia and alpha motor neurons [12, 23, 24, 26, 27]. The effects of acupoints DN on spasticity as investigated in this study might also be explained by various activation/deactivation changes in the regions of the brain associated with sensation, vision, and motion [28].

A case study using functional magnetic resonance imaging (fMRI) in a patient with chronic stroke found that a single session of DN applied to LI4 led to increased activation in both the affected and unaffected primary motor cortices, the affected primary somatosensory cortex, and the affected supplementary motor area. These changes were associated with improvements in wrist flexor spasticity, active wrist extension, and hand function [29]. Another case study applied a single session of DN to the spastic brachialis muscle of a patient with chronic stroke and used electroencephalogram (EEG) with a complex network approach to assess DN's effects on the central nervous system. The authors reported improvements in brain activity and changes in local brain network parameters in the delta, theta, and alpha frequency bands, trending toward patterns observed in healthy control [30].

In this study, both active and passive wrist extension ROM improved after DN, and improvement enhanced at 1 week follow-up. However, the wrist extension ROM decreased at 6-week follow-up when compared to those at 1-week follow-up (↓10°) but still better than baseline (active ROM 0° vs. 35°; passive ROM 90° vs. 80°). Improvements in wrist extension ROM may be explained by improvements in spasticity of wrist flexors. The fact that both wrist active and passive ROM improved after DN suggests that the intervention may have had a more holistic and positive effect on the wrist muscles' function. This indicates that DN by simultaneously reducing spasticity and improving muscle flexibility, and neuromuscular control contributed to improved wrist active ROM. Improvement in active ROM suggests that the patient was better able to voluntarily control the wrist extensors, which is a sign of motor recovery. Improvements in both wrist active and passive extension are aligned with those reported in previous studies [31, 32].

Both active and passive ROM remained better than baseline at 6 weeks—even with a decline from the 1-week follow-up. This indicates that DN of LI4 and TE5 acupuncture points has a lasting effect in reducing spasticity and improving muscle function. This finding is especially important in patients with poststroke spasticity, where improving active ROM can lead to greater independence. Even though DN may not have fully maintained the peak improvement achieved at 1 week, it might be a valuable tool in the short to medium term for improving spasticity, muscle flexibility, and voluntary control. The fact that the patient maintained better ROM at follow-up than baseline suggests a positive trend. Follow-up DN at regular intervals might help sustain the benefits over the longer term. Also, the effects might be supplemented with exercise therapy and neurorehabilitation strategies for maximizing the longer-term benefits [33]. Therefore, studies are required to evaluate whether the periodic DN combined with functional exercise therapy could maintain both wrist active and passive ROM in poststroke patients with spasticity.

The similarity in trends for both wrist active and passive ROM is interesting. It implies that the mechanisms driving the improvement are likely related. As DN reduces spasticity, it allows for better relaxation of wrist spastic flexors and lengthening (passive ROM), which then translates into better voluntary control and coordination (active ROM). This could suggest that DN may address both the muscle spasticity and voluntary neuromuscular control, which is crucial for stroke rehabilitation.

This study indicated small improvements in the motor function of the upper limb reflected in the Fugl-Meyer Assessment Score, similar to other studies which found sensorimotor changes after DN [34]. The literature indicates that the acupuncture can improve the limb function after stroke by various mechanisms [26]. Small improvements in motor function observed in this case with chronic stroke might be due to the technique used, applying DN with manipulation of needle in LI4 and TE5 acupoints. Another possible reason might be that reduction in spasticity is not necessarily translated to motor function, and improvement in motor function requires task-related exercises. A systematic review and meta-analysis on the efficacy of acupuncture for spasticity paralysis in patients poststroke concluded its pronounced effects in improving limb motor function and daily life activities compared to conventional regimens [35]. However, a recent study demonstrated that the combined DN and exercise therapy did not provide additional improvements in motor function compared to only DN [36]. In this study, DN was applied to the acupuncture points with manipulation, and no significant changes were observed in the Fugl-Meyer Assessment Score. Studies with this technique combined with the task-oriented exercises are needed to investigate whether adding the exercise therapy would yield additional benefits in motor function.

This case report provides initial findings on the use of the DN technique on acupoints in a patient with chronic stroke and consistent with the literature [9, 21, 34, 37, 38] found improvements in spasticity, wrist AROM, and PROM. Further high-quality investigations with longer-term follow-up are needed to demonstrate the application of DN on acupuncture points and in combination with exercise therapy for the treatment of motor function poststroke. Future studies should clarify the complementary value of DN on acupuncture points compared to conventional treatment.

## Figures and Tables

**Table 1:** The Modified Modified Ashworth Scale of spasticity [13].

Grade	Definition
0	No increase in muscle tone
1	Slight increase in muscle tone, manifested by a catch and release or by minimal resistance at the end of the range of motion when the affected part is moved in flexion or extension
2	Marked increase in muscle tone, manifested by a catch in the middle range and resistance throughout the remainder of the range of motion, but affected part easily moved
3	Considerable increase in muscle tone, passive movement difficult
4	Affected part(s) rigid in flexion or extension

*Note:* Nakhostin Ansari et al. Development of the Persian version of the Modified Modified Ashworth Scale: translation, adaptation, and examination of interrater and intrarater reliability in patients with poststroke elbow flexor spasticity. Disabil rehabil 2012 34, no. 21:1843–1847.

**Table 2:** Results of outcome measures at time points.

Outcome measures	Baseline (T0)	After 3 sessions (T1)	1-week follow-up (T2)	6-week follow-up (T3)
Wrist flexor	2	0	0	0
MMAS score				

*Wrist extension*				
AROM (degree)	0	10	45	35
PROM (degree)	75	82	90	80

FMA total score	38	39	45	38
FMA-UE	23	23	24	22
FMA wrist	0	0	5	0
FMA hand	10	11	11	10
FMA coordination-speed	5	5	6	6

Abbreviations: AROM, active range of motion; FMA, Fugl-Meyer assessment; MMAS, Modified Modified Ashworth Scale; PROM, passive range of motion.

## Data Availability

The data generated during the current case study are available on reasonable request.
